# Climate Change Cannot Explain the Upsurge of Tick-Borne Encephalitis in the Baltics

**DOI:** 10.1371/journal.pone.0000500

**Published:** 2007-06-06

**Authors:** Dana Sumilo, Loreta Asokliene, Antra Bormane, Veera Vasilenko, Irina Golovljova, Sarah E. Randolph

**Affiliations:** 1 Department of Zoology, University of Oxford, Oxford, United Kingdom; 2 Centre for Communicable Diseases Prevention and Control, Vilnius, Lithuania; 3 State Agency ‘Public Health Agency’, Riga, Latvia; 4 National Institute for Health Development, Tallinn, Estonia; London School of Hygiene & Tropical Medicine, United Kingdom

## Abstract

**Background:**

Pathogens transmitted by ticks cause human disease on a greater scale than any other vector-borne infections in Europe, and have increased dramatically over the past 2–3 decades. Reliable records of tick-borne encephalitis (TBE) since 1970 show an especially sharp upsurge in cases in Eastern Europe coincident with the end of Soviet rule, including the three Baltic countries, Estonia, Latvia and Lithuania, where national incidence increased from 1992 to 1993 by 64, 175 and 1,065%, respectively. At the county level within each country, however, the timing and degree of increase showed marked heterogeneity. Climate has also changed over this period, prompting an almost universal assumption of causality. For the first time, we analyse climate and TBE epidemiology at sufficiently fine spatial and temporal resolution to question this assumption.

**Methodology/Principal Finding:**

Detailed analysis of instrumental records of climate has revealed a significant step increase in spring-time daily maximum temperatures in 1989. The seasonal timing and precise level of this warming were indeed such as could promote the transmission of TBE virus between larval and nymphal ticks co-feeding on rodents. These changes in climate, however, are virtually uniform across the Baltic region and cannot therefore explain the marked spatio-temporal heterogeneity in TBE epidemiology.

**Conclusions/Significance:**

Instead, it is proposed that climate is just one of many different types of factors, many arising from the socio-economic transition associated with the end of Soviet rule, that have acted synergistically to increase both the abundance of infected ticks and the exposure of humans to these ticks. Understanding the precise differential contribution of each factor as a cause of the observed epidemiological heterogeneity will help direct control strategies.

## Introduction

There is as yet no good explanation for the remarkably rapid increase in the incidence of tick-borne encephalitis (TBE) over the past one to two decades in many parts of Europe. Before any explanation can be found it is essential to define the problem accurately. Simple national statistics hide considerable variation in the timing and extent of the increases at finer spatial scales that makes many of the more obvious explanations unlikely to be wholly correct. Climate change is all too often proposed as the cause of the observed upsurges of many different tropical and temperate vector-borne diseases [*inter alia* 1]–[5], including TBE [6]–[8]. Changes in surveillance and diagnostic practices are commonly given as reasons to dismiss the changed incidence as no more than an artefact. While changes in climate and public health have undoubtedly occurred over recent decades, such changes are likely to apply more or less simultaneously across wide geographical and national regions, respectively, whereas changes in TBE epidemiology show marked spatio-temporal variation and incidence has not in fact increased everywhere. Furthermore, in Latvia incidence has decreased again since 1998.

TBE is a typical zoonosis caused by a virus transmitted by ticks, principally *Ixodes ricinus* and *I. persulcatus*, amongst small rodents, principally mice of the genus *Apodemus* [9]. Ticks feed only once per life stage, as larvae, nymphs and adults. Only rodents are competent transmission hosts for TBE virus, and the period of rodent infectivity is limited to a few days. Furthermore, only larvae and nymphs habitually feed on rodents, so that natural amplification of TBE virus to allow persistent cycles depends on transmission from the relatively few infected nymphs to the much more abundant infectible larvae (which then moult to infected, host-seeking nymphs up to one year later) [10]. This transmission route demands that larvae and nymphs feed together on individual rodents (so-called co-feeding), which occurs to different degrees according to the temperature-driven patterns of seasonal activity of each tick stage. Tick survival from stage to stage depends on habitat structure that determines moisture conditions on the ground and host availability. Together these factors determine the focal distribution of TBE [11], but have not been shown to determine incidence.

The risk of exposure of humans to infected tick bites therefore depends on a wide range of environmental factors, both abiotic (climate and landscape features) and biotic (tick and host distributions and abundance), and on socio-economic factors affecting human behaviour. As the tip of the zoonotic iceberg expands, explanations should identify factors that determine growth in the submerged bulk (due mostly to biological factors that determine the virus transmission potential) and those that cause an increase in *relative* exposure above the surface (due more to factors that determine human contact with infected ticks, and also to human susceptibility to infection). TBE and Lyme borreliosis (LB), the latter caused by spirochaete bacteria vectored by the same tick species, are the most significant vector-borne diseases in Europe. Whereas LB, however, has been recorded non-systematically only since the mid 1980s, cases of TBE have been registered systematically for the past 30–50 years. TBE therefore offers a perfect case study of the diverse impact of humans, both direct and indirect, on zoonotic risk.

As a first step towards seeking explanations for the geographical mosaic of TBE epidemiology seen across Europe [12]–[15], this study focuses on the Baltic countries. From north to south, Estonia, Latvia and Lithuania occupy the northeast corner of Europe on the eastern coast of the Baltic Sea. In the early 1990s, at the time of independence from Soviet rule, they each experienced more dramatic increases in national TBE incidence than anywhere else in Europe apart from Poland, raising their annual incidence (from c.0.5–7 to 11–54 cases per 100,000 population) to the highest levels in Europe, matched only by Slovenia. Despite the small size of these countries (45,200–65,300 km^2^), the pattern of TBE increase in individual ‘counties’ (15 in Estonia, 26 in Latvia and 44 in Lithuania) was markedly non-uniform, but typically extremely sudden, rising by up to one, even two, orders of magnitude within a single year relative to the previous 10-year mean level. Furthermore, the degree of increase was not always correlated with previous high-risk conditions [16] (see below), and the year of first increase ranged from 1990 to 1998 for different counties. Within the Baltic countries there is thus an ideal opportunity to test whether climate has changed at the right time, in the right places and in the right way to account for the spatially variable abrupt discontinuities in TBE incidence. This paper presents the results of this test and, following a negative result, proposes an alternative hypothesis to account for the observed TBE epidemiology.

## Materials and Methods

Annual TBE case numbers registered by the patients' place of permanent residence (county or large city) for the period 1970–2004 were obtained from national Public Health Agencies in Estonia, Latvia and Lithuania. More detailed data from Latvia and Lithuania on the place of infection, where known, indicated that c.84% of infections were acquired within the patients' home county. Human population data, available from National Statistical Offices from 1970, were used to convert case numbers into incidence per 100,000 of the population.

Daily or 10-day-mean (dekadal) records of minimum and maximum air temperature, precipitation (mm) and snow cover (cm) recorded at 6–8 ground stations per country were acquired from national Meteorological Agencies (see map). These stations were selected to be as close as possible to suitable tick habitat and to be least influenced by heat sources such as airports, factories, etc. They were also selected to represent observed contrasting epidemiological patterns and also those parts of Estonia and Latvia where either *I. ricinus* ticks occur alone or with *I. persulcatus* (in eastern counties) (see map). Where necessary, the daily records were converted into means for each of three dekads per month to capture temporal changes at a sufficiently fine resolution to match tick biology.

Discontinuities in both the epidemiological and meteorological data were identified by computing generalized distance time series [17]. The generalized distance *D* ^2^ is a boundary-locating algorithm designed to detect sudden changes in mean values over a given segment of length 2*h*+1 of the temporal window being considered (here 1970–2004), where *h* is a specified number of time steps. *D* ^2^ was calculated for every time step *j* (i.e. year) for each set of observations, TBE incidence, daily maximum temperature, rainfall and snow cover, as follows:
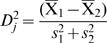
 where X̅_1_ is the mean of observations from *X_j−h_* to *X_j_*, X̅_2_ is the mean of observations from *X_j_* to *X_j+h_*, and the denominator is simply the sum of the variances (*s* ^2^) calculated over each of the two parts of the temporal window around *X_j_* [18]. To accommodate the relatively short period of observations available, 35 years, a value of *h* = 6 years was selected; short enough to yield a time series over a meaningful part of this period, but not so short as to respond too sensitively to short-term fluctuations. With smooth data, *D* ^2^ peaks at any discontinuity, whether an increase or a decrease, but given the highly non-smooth nature of these data, the precise year of change indicated by the *D* ^2^ statistic does not always coincide with a significant change relative to the full data series [18]. The statistic was therefore interpreted with reference to absolute values of the raw data, and in conjunction with other statistics such as anomalies from the overall means and simple Student's *t*-tests applied to the mean values either side of any discontinuity.

## Results

### Changes in TBE incidence 1970–2006

**Figure 1:**
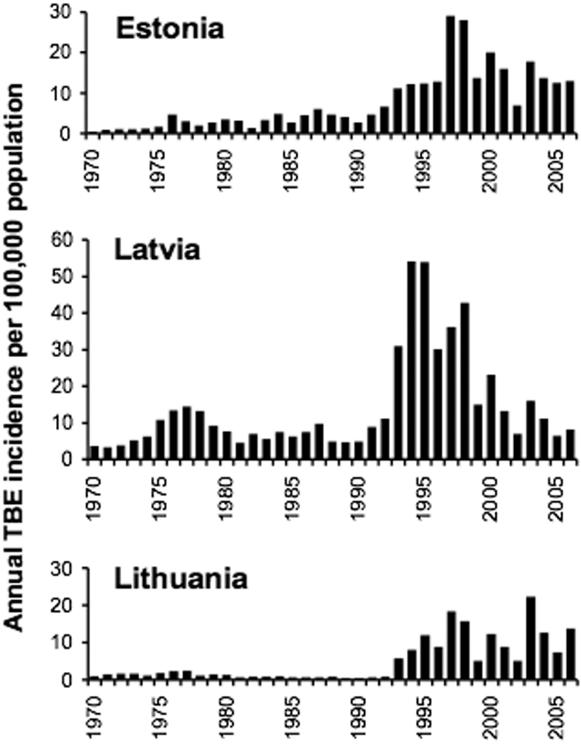
National annual TBE incidence per 100,000 population, 1970–2006 in Estonia, Latvia and Lithuania.

Nationally, the Baltic countries all show similar patterns of change in TBE incidence over the past 35 years (Fig. 1), despite the different mean levels per country. Both Latvia and Lithuania showed somewhat higher incidence in the late 1970s than throughout the 1980s, but in Estonia the modest increase in the late 1970s was maintained through the 1980s. All countries suffered an abrupt upsurge in 1993, when, relative to the previous 10-year means, incidence increased 2.6-fold in Estonia, 4.5-fold in Latvia and 13.8-fold in Lithuania and continued to increase over the following 5 years. Latterly, the incidence in Latvia has reverted to levels similar to the late 1970s, but no such decline has occurred in Estonia or Lithuania.

The synchrony of this upsurge does not, however, apply to all counties within each country. We are here concerned with the start point of the upsurge, but the peak value of the *D* ^2^ statistic indicates the largest discontinuity, with its timing much affected by isolated events before and after. The start point, therefore, is taken as the year in which TBE incidence first showed a 2-fold increase above the previous 10-year mean and also exceeded any previous level in any one year during those 10 years. It varied from 1990 to 1998, although it occurred in 1993 or 1994 in 50% of counties (Table 1). There was no consistent change in 10 (12%) counties, either because incidence was already high and remained so (1 county in Estonia and 2 in Latvia) or because zero or only 1 or 2 cases occurred (1 county in Latvia and 6 in Lithuania). One might expect the upsurge to occur later where TBE incidence was lower, but there was no correlation between the timing of the upsurge and the mean annual incidence over the decade before the upsurge to support this trivial explanation (*R* ^2^ = 0.0001, 0.041, and 0.065, *p*>0.05, n = 26, 15 and 44 for Latvia, Estonia and Lithuania, respectively).

**Table 1:** Years in which the upsurge in TBE started in each county within the Baltic countries.

	No. counties with TBE upsurge starting in each year		
	Estonia	Latvia	Lithuania
1990		1	
1991	2	2	1
1992	2	3	2
1993	1	12	13
1994	4	3	10
1995	1	1	5
1996	2	1	2
1997	1		4
1998	1		1
no consistent change	1	3	6

For definition of upsurge, see text.

**Figure 2:**
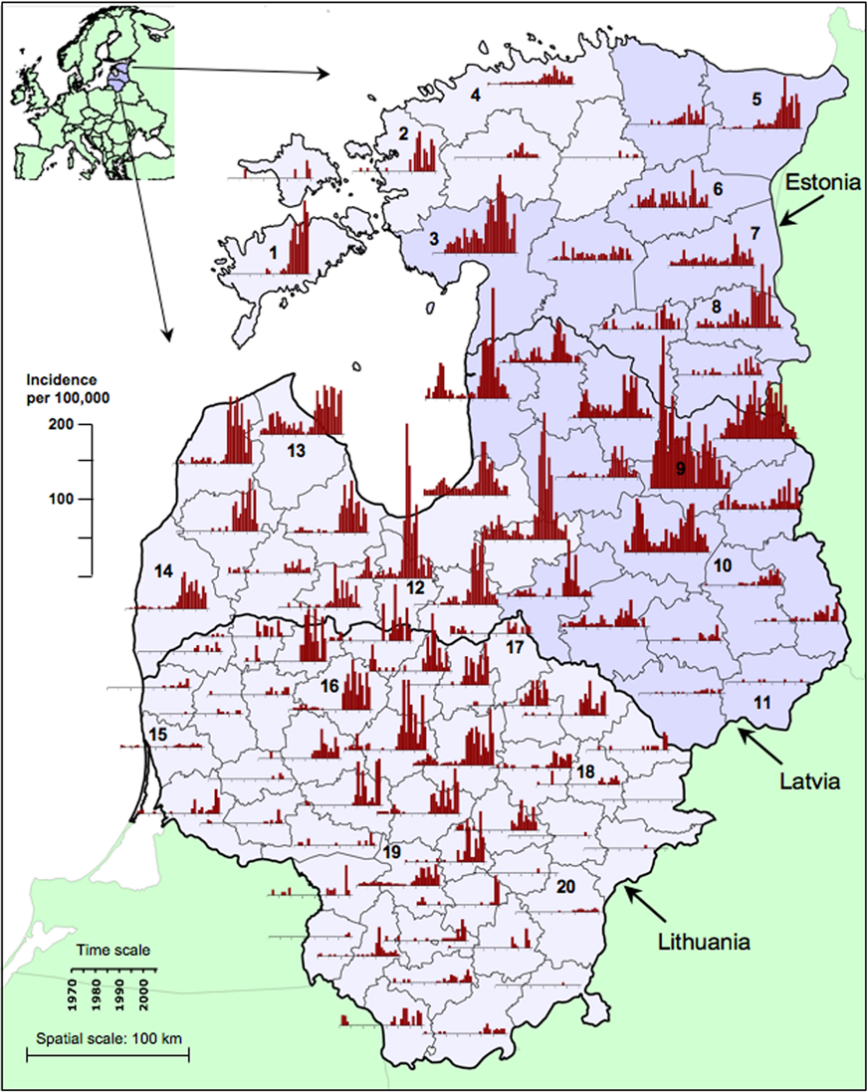
Annual TBE incidence per 100,000 population (*y*-axis) in each county of Estonia, Latvia and Lithuania, 1970–2004 (*x*-axis). *I. ricinus* is present throughout and *I. persulcatus* occurs in the darker shaded areas of Estonia and Latvia. The numbers refer to the 20 counties for which meteorological data have been analyzed: Estonia, 1 Saaremaa, 2 Läänemaa, 3 Pärnumaa, 4 Harjumaa, 5 Ida-Virumaa, 6 Jõgevamaa, 7 Tartumaa, 8 Võrumaa; Latvia, 9 Gulbene, 10 Rezekne, 11 Kraslava 12 Jelgava, 13 Talsi, 14 Liepaja; Lithuania, 15 Klaipeda, 16 Siauliai, 17 Birzai, 18 Utena, 19 Kaunas, 20 Vilnius.

The following notable points illustrate the variability in the changing patterns of annual TBE incidence per county, shown in Fig. 2. Many counties registered zero or extremely few cases before the early 1990s, after which either the incidence rocketed or only a few cases appeared for several years. The degree of increase in each county (measured as the ratio of the mean for 5 years post-increase to the mean for 10 years pre-increase in counties with at least some cases registered before the upsurge) ranged from 2.5–74 in Estonia to 3–34 in Latvia and 8–706 in Lithuania, and was not related to the incidence prior to 1980 (i.e. incidence independent from that used to estimate the proportional increase). In Latvia, of the eight counties with the highest TBE incidence (>10/100,000) prior to 1980, six followed the common pattern of reduced incidence during the 1980s before an upsurge to incidences equal to or usually higher than the historic levels; exceptions were Gulbene county (numbered 9 in Fig 2), where annual incidence was usually lower in the mid-1990s than in the 1980s, and Aluksne county (immediately northeast of Gulbene), where incidence increased during the 1980s but very little thereafter. These variable patterns were not related to the presence of the second species of tick, *I. persulcatus* (Fig. 2).

### Changes in climate 1970–2004

Daily minimum and maximum temperatures were highly correlated. As the purpose of this analysis is to identify discontinuities in climatic conditions that may explain the discontinuities in TBE epidemiology in the Baltic countries during the period 1970–2004, the results will be presented for maximum (i.e. day-time) air temperature because there exists some knowledge of the threshold daily maximum temperature necessary for *I. ricinus* host-questing activity [10], [19], [20], while the biological relevance of minimum temperatures, presumed to be their impact on tick mortality, has yet to be quantified.

**Figure 3:**
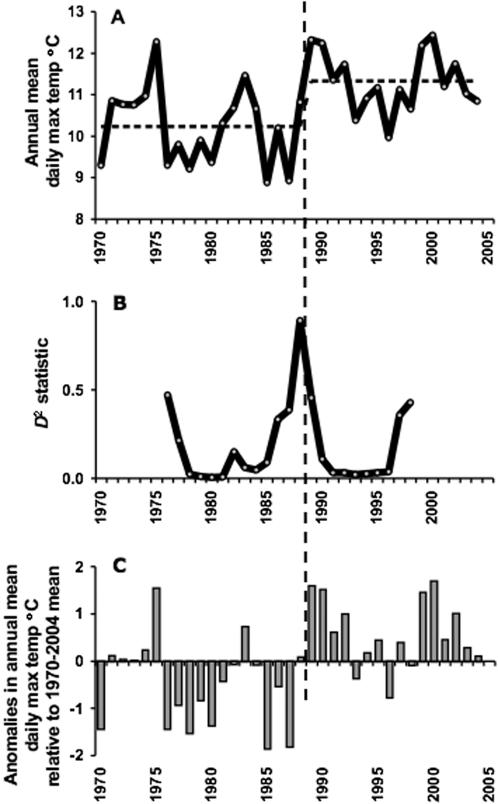
Annual mean daily maximum air temperature recorded at the meteorological station at Jelgava, Latvia. A) Annual mean daily maximum air temperature, with mean temperatures 1970–1988 and 1989–2004 indicated by the horizontal dotted line. B) Generalized distance statistic *D* ^2^ indicating the major discontinuity through time. C) Anomalies from the mean level 1970–2004. The vertical dashed line indicates the timing of the marked increase from 1989 onwards (see text).

Annual mean daily maximum air temperatures (AMDMAT) for the period relevant to TBE epidemiology in the Baltic countries showed a step increase from 1989 onwards (Fig 3 for an example at Jelgava, Latvia). The *D* ^2^ statistic (Fig 3b) peaked at 1988, reflecting the increase relative to the previous three cool years, but that merely brought the temperature very close to the overall mean for 1970–2004 mean. It was not until 1989 that the temperature consistently exceeded previous levels. There was no discernible trend either before or after this date, as shown by the fluctuations about the mean level for each period (horizontal dotted, Fig 3a), and by the switch in direction, but no change in magnitude, of the anomalies from the overall mean level for 1970–2004 (Fig. 3c). At the 20 sites investigated, the increase in the mean level of the AMDMAT between 1970–1988 and 1989–2004 was 0.94–1.38°C and was maximally statistically significant at this breakpoint in all cases (Student's *t* = 3.05–5.08, df = 33, p<0.005) (Table S1).

**Figure 4:**
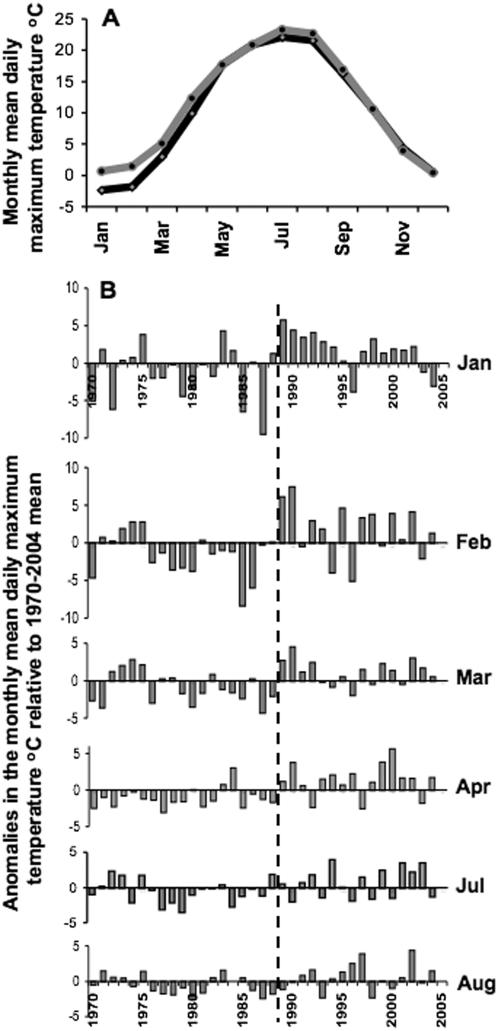
Monthly mean daily maximum air temperature recorded at the meteorological station at Jelgava, Latvia. A) Monthly mean daily maximum air temperature 1970–88 (black line) and 1989–2004 (grey line). B) Anomalies from the mean level 1970–2004 for months that show a significant increase in temperature from 1989, indicated by the vertical dashed line.

Annual mean conditions serve to focus attention on the discontinuous nature of past temperature increases in Europe, but are irrelevant to any type of free-living organism that lives under seasonally variable daily thermal conditions. Anomalies from the overall mean levels of each monthly mean daily maximum air temperatures (MMDMAT) for 1970–2004 reveal that increases in winter-early spring temperatures contributed most to the change in AMDMAT (Fig. 4). At all sites, during January to April an abrupt and statistically significant switch from predominantly negative to predominantly positive anomalies occurred in 1989. A similar temperature increase occurred in July at 14 of the 20 sites, in August at 16 sites and in September at 4 sites (Table S1), but in these months the most marked switch occurred in 1991 rather than 1989 (Fig. 4). On the other hand, mean levels of MMDMAT were marginally lower for 1989–2004 than 1970–88 in May (at 12 sites), June (5), October (7), November (19) and December (9).

**Figure 5:**
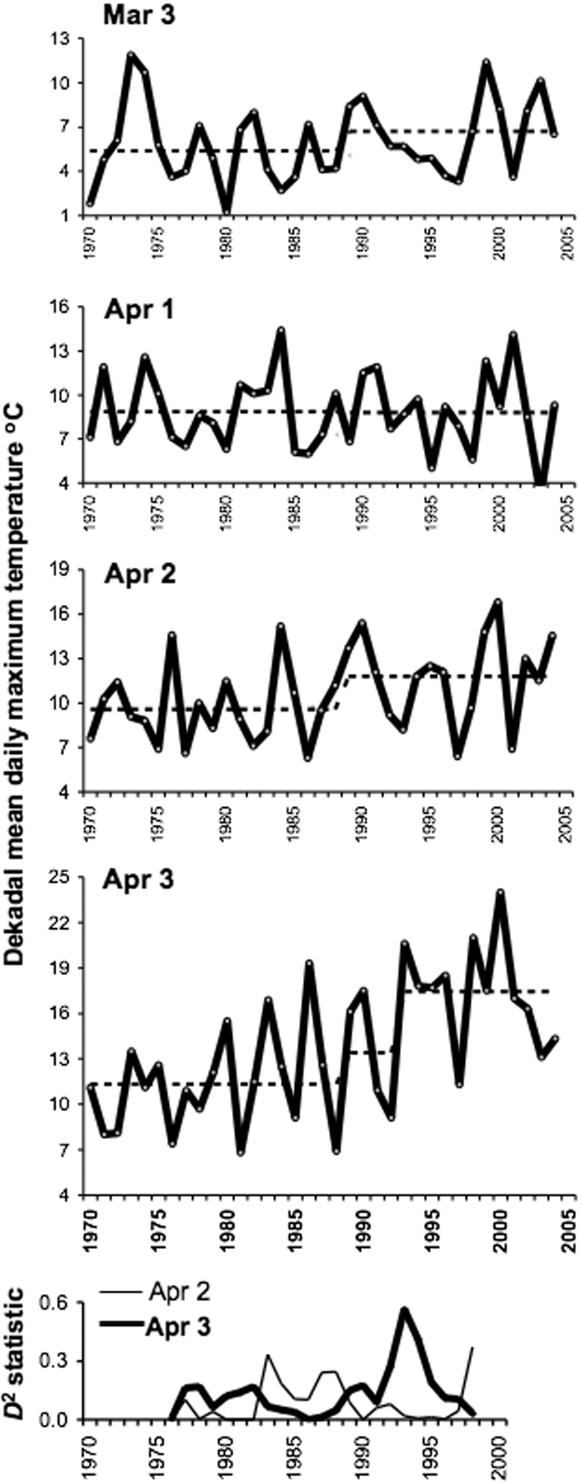
Dekadal mean daily maximum air temperature for 21.March-30. April recorded at the meteorological station, Jelgava, Latvia. The horizontal dotted lines indicate mean temperatures for 1970–1988 and 1989–2004, except for the 3^rd^ dekad of April, where the more marked increase from 1993 is also highlighted; this discontinuity is confirmed by the *D* ^2^ statistic peak (bottom graph).

**Figure 6:**
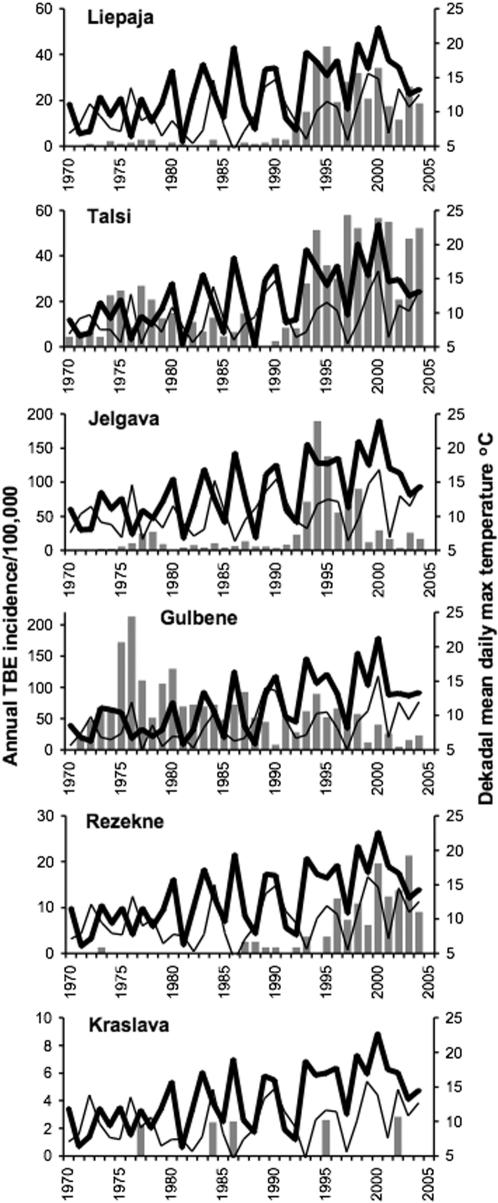
TBE incidence (grey bars), and daily maximum temperature for 2nd (thin line) and 3rd (heavy line) dekads of April. Examples from meteorological stations in each of six counties in Latvia.

Even these monthly means hide details at time scales relevant to tick biology. Increased air temperatures during the winter and early spring are irrelevant to ticks that are inactive under such cold conditions (see mean monthly temperatures 1989–2004, Table S1) undergoing overwinter diapause at soil level, commonly beneath snow cover. The critical time for ticks in the Baltic countries is April, when temperatures start to cross the threshold for activity, and the time scale appropriate to the onset of activity by ticks of different stages with different temperature sensitivities is dekadal (10-day) rather than monthly. For March and April, there was no significant step increase in 1989, nor any other trend, in dekadal mean daily maximum air temperatures (DMDMAT) for the third dekad of March or the first dekad of April, but there was for the first two dekads of March (not shown) and the last two dekads of April (Fig. 5 and Table S1). Furthermore, exclusive to the third dekad of April, there was a much more significant step increase in 1993 than in 1989, clear from both the absolute mean temperatures and the *D* ^2^ statistic: at Jelgava, for example, the DMDMAT was on average 2°C higher for 1989–92 than for 1970–88, but 4°C higher for 1993–2004 than for 1989–92. This pattern was consistent for all sites in the Baltics, although least marked in Estonia (Table S1), with the effect that in all years since 1993 (except for 1997 at 9 sites throughout the region, plus 2003 at 2 sites in Estonia, and 6 years at the southern tip of the island of Saaremaa, Estonia) DMDMAT for the third dekad of April has been consistently above 10°C (Fig. 6: data for Latvia shown, data for Estonia and Lithuania not shown). This is the approximate threshold for larval *I. ricinus* activity and therefore permits co-feeding of larvae with nymphs (see Introduction). In years prior to 1993, and in dekads prior to the end of April, DMDMAT commonly fell between 7°C (the threshold for nymphal activity) and 10°C (Figs. 5, 6), therefore permitting nymphs to feed in the absence of co-feeding larvae. There was no spatial variation in this pattern of temperature change during the spring, nor at any other time of the year, that could be related to the variable patterns of increase in TBE incidence in each county. Conversely, exhaustive efforts failed to identify any relationship between the inter-annual variation in any aspect of thermal conditions and the highly variable annual TBE incidence per county throughout the Baltic countries, either during the recent decade of high incidence or for the whole period 1970–2004.

**Figure 7:**
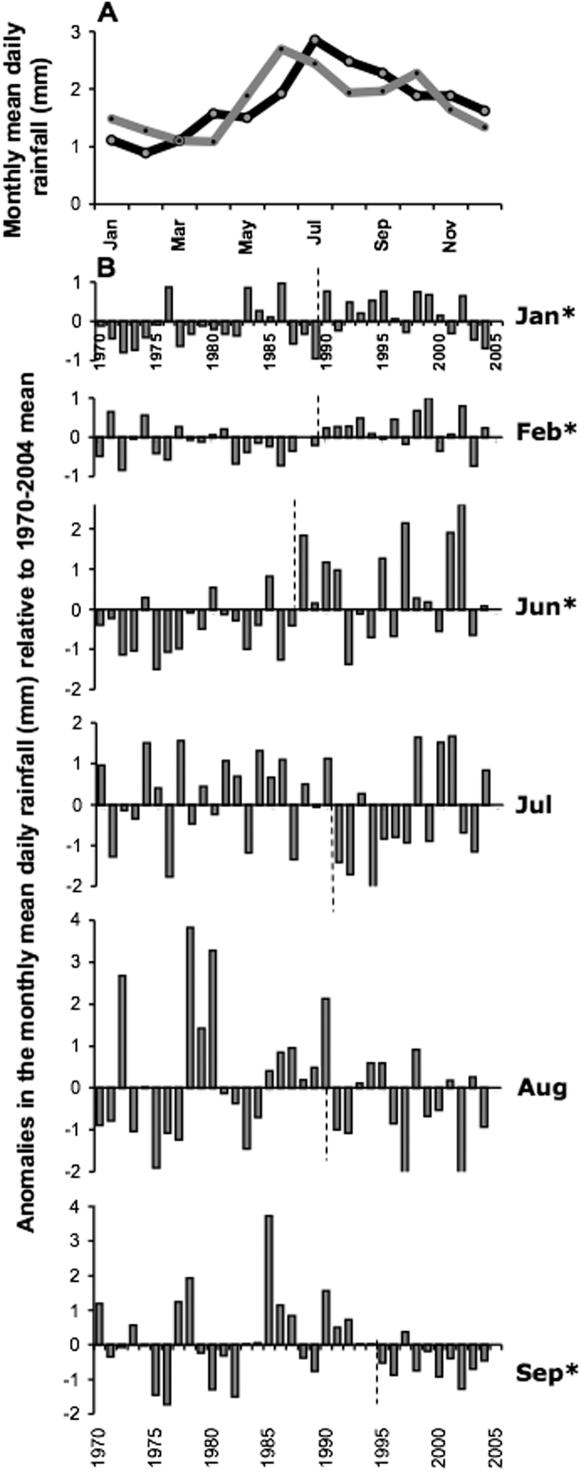
Daily rainfall (mm) recorded at the meteorological station at Jelgava, Latvia. A) Monthly mean daily rainfall 1970–89 (black line) and 1990–04 (grey line). B) Anomalies from the mean level 1970–2004 for months that show a (significant, marked with an asterisk) change after the year indicated by the vertical dashed line (see Table 2).

Two aspects of precipitation are relevant to tick-borne diseases: rainfall, especially during the summer when ticks may die of desiccation, and snow cover as a protective blanket for ticks undergoing diapause at ground level. Throughout the region, there were small but statistically significant changes in monthly rainfall most commonly starting around 1990, although the precise timing of the maximally significant discontinuities varied geographically and from month to month (Fig. 7, Table 2). Most consistent was an *increase* in rainfall during February from 1990 or a little earlier (also in January and March in some parts, notably Estonia) (Fig. 7, Table 2). At about the same time, June also became wetter in many places. Although rainfall in the late summer was most variable (see anomalies in Fig. 7b), a statistically significance *decrease* was commonly recorded in July from 1991 in Latvia, and in September from 1995 in Latvia but 1989 in Estonia. Overall, significant rainfall changes were less common in Lithuania.

**Table 2:** Years of maximally statistically significant discontinuities in rainfall and snow cover recorded at sites in the three Baltic countries.

Rainfall		Year of maximally significant discontinuity											
	County	Jan	Feb	Mar	Apr	May	June	July	Aug	Sep	Oct	Nov	Dec
**Latvia**	Jelgava	***1990***	***1990***		1990		***1988***	1991	1991	**1995**			
	Dagda		***1990***	***1988***			*1991*	**1991**					
	Gulbene	***1990***	***1990***				*1988*			**1995**	***1991***		
	Liepaja		***1990***					**1991**					
	Rezekne		***1990***					**1991**		**1995**			
	Talsi		***1990***				***1990***						
**Lithuania**	Birzai		***1990***				***1991***				***1989***		
	Kaunas		***1989***										
	Klaipeda		***1987***					**1991**				**1993**	
	Siauliai		***1990***		1987						***1989***	**1992**	
	Utena		***1989***		**1987**		***1987***		**1987**		*1989*		
	Vilnius		***1989***										
**Estonia**	Harjumaa		***1990***							**1989**			
	Läänemaa		***1987***						**1994**	**1993**			
	Pärnumaa	***1990***	***1991***	***1992***			*1988*						
	Saaremaa		***1989***							**1989**			
	Jõgevamaa		***1989***	***1988***			*1989*			**1989**			
	Ida-Virumaa		***1989***				***1989***			1989			
	Tartumaa	***1990***	***1990***	***1990***			***1989***						
	Võrumaa	***1990***	***1990***	***1988***									**1989**
**Snow cover**													
**Latvia**	Jelgava												
	Dagda		**1988**	**1989**							***1990***		
	Gulbene		**1988**	**1989**							*1990*		
	Liepaja												
	Rezekne	**1988**	**1988**	**1989**									
	Talsi		**1988**									1990	
**Lithuania**	Birzai		**1988**								***1988***	***1987***	
	Kaunas		1988										
	Klaipeda												
	Siauliai		**1988**								***1992***	***1988***	
	Utena		**1988**		*1986*								
	Vilnius	1988	**1988**		*1986*						***1991***	***1988***	

Italics indicate an increase, non-italics indicate a decrease. Bold type, p<0.05, regular type, p<0.1 for Student's *t*-test on mean values for periods either side of the discontinuity (within the period 1970–2004).

Snow cover in Latvia and Lithuania (records not available for Estonia) melted by the end of March, with small amounts sometimes persisting into April, and reappeared in very small amounts (typically 1–2 cm) during October and November. Snow cover in January–March was markedly deeper (commonly 10–40 cm) during 1976–87, decreasing significantly from 1988 except in counties of low snow fall on the west coast, but appearing slightly earlier in late autumn in some years at some places (Table 2).

Again it proved impossible to relate these spatial and temporal patterns of changes in either rainfall or snow cover to the epidemiology of TBE.

## Discussion

### Is the upsurge of TBE in the Baltic countries an artefact?

The heterogeneity (Fig 2) in the timing and degree of changes in the incidence of TBE between counties within each country is inconsistent with increased incidence being due to any national changes in public health practices. Furthermore, exhaustive searches through archives (paper and electronic) and face-to-face discussions with public health and medical personnel active both before and after the up-surge in cases of TBE, have provided convincing evidence (D. Sumilo 2006, *DPhil thesis*, Oxford University) that any changes in public health activities may possibly have exaggerated the up-surge a little, but cannot explain its full extent. Serological diagnosis of TBE was in place from the 1970s and all notified cases were required to be laboratory confirmed. The ELISA method of diagnosis was introduced in 1993 in Estonia and Latvia, but not until 1994 in Lithuania, and ran in parallel with the earlier haemaglutination inhibition method for a few years. There is consistent denial, by both Baltic and Russian professionals, of any suppression of TBE case reports under Soviet rule. The conclusion is that the upsurge in TBE incidence was real and requires an explanation.

### The explanatory power of climate change

From this analysis it is clear that aspects of climate that are relevant to tick-borne encephalitis transmission have changed over the past 35 years. It is equally clear, however, that although these changes may have provided a generally more permissive climatic background for TBEv transmission, they are too similar throughout the Baltic countries to account for the highly heterogeneous patterns of change in TBE incidence since the early 1990s. Spring temperatures have increased significantly, but as a step increase in 1989 rather than in the gradual way normally implied by seeking trends and superimposing a best-fit curve [21], [22]. Our method of applying a statistic designed to identify discontinuities, and then cutting the 35-year time series at the identified point of discontinuity, not only reflects the observations, but also coincidentally focuses attention on the critical period in relation to TBE epidemiology. It is not the purpose of this paper to address the larger picture of climate change (nor to provide explanations for the meteorological observations), but the conclusions are not diminished by extending the observations back to the 1960s or to other parts of Europe; a step increase is apparent in temperature records for most of Europe, with fluctuations but no consistent change prior to the end of the 1980s (http://ipcc-ddc.cru.uea.ac.uk). Estimates of the mean increase in temperature are given (Table S1) to illustrate the regional uniformity and seasonality of the warming.

The observed temperature changes from 1989 could have enhanced the transmission potential for TBEv by permitting earlier onset of tick activity in the spring. This could have been exacerbated from 1993 onwards when mean daily maximum temperatures at the end of April consistently exceeded the threshold for larval *I. ricinus* activity (c.10°C [10]), thereby reducing the delay between the onset of conditions permissive for this stage and for nymphal *I. ricinus* (7°C [10], [19], [20]). Changes such as this, which would increase the seasonal synchrony consistently between these tick stages to permit virus transmission from infected nymphs to infectible larvae co-feeding on rodents, would be expected to increase the prevalence of infection in ticks [11]. Temperature thresholds for *I. persulcatus* activity are unknown. A short delay of a few years between this climate change and the upsurge of TBE incidence might be expected, as the majority of larvae feeding and acquiring virus in one year do not give rise to infected host-questing nymphs until the following year [23] and infection prevalence in the tick population could take time to build up. The timing of the upsurge is consistent with this only in some counties (Table 1), and its variability cannot be accounted for by the uniform timing of the temperature changes.

The same sort of conclusion arises from considering daily minimum temperatures that have changed in parallel with maximum temperatures. In this case, the biological significance of warming may lie in reduced tick overwinter mortality. As would be expected from the geographical distribution of *I. ricinus* as far north as c.65°N in Sweden [24] and *I. persulcatus* across northern Russia, both tick species are adaptively cold-hardy, but nevertheless *I. ricinus* has been observed to suffer increased mortality when exposed experimentally to increasingly sub-zero temperatures below −10°C [25]. The natural level of mortality, however, and its sensitivity to natural winter conditions beneath snow cover are not known. In any case, whatever the improvement in overwinter survival caused by the step increase in minimum temperatures from 1989 onwards, the pattern of increased minimum temperature was similar throughout the region. Furthermore, the different range of these two tick species suggests differential sensitivities to thermal conditions, but there is no consistent difference in patterns of TBE change in counties dominated by either *I. ricinus* or *I. persulcatus* in Estonia and Latvia (Fig. 2).

Decreased rainfall in mid to late summer could adversely affect ticks, causing increased desiccation stress, but because this decrease occurred during the wettest season of the year, and also commonly followed an increase in rainfall in June, it is unlikely that conditions became limiting for either survival or questing by ticks. The observed 25% decrease in rainfall may, however, have allowed an increase in people's outdoor recreation, which is known commonly to include visits to tick-infested forests (see below).

In conclusion, in the absence of any identified differences *between* counties, climate changes alone do not offer a sufficient explanation for the observed spatio-temporal heterogeneity in epidemiological changes.

### An alternative hypothesis to explain the heterogeneous epidemiology of TBE

**Figure 8:**
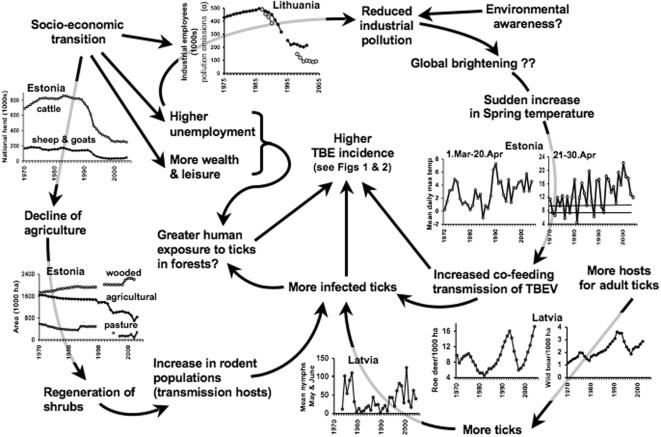
Hypothetical explanation for the epidemiology of TBE in the Baltic countries. Examples of data from Estonia, Latvia and Lithuania indicate some factors that may act independently but synergistically to cause the emergence of tick-borne diseases.

Considerable human-induced changes in environmental factors other than climate have also occurred in the Baltic countries. There is quantified evidence of indirectly linked changes in several different abiotic, biotic and non-biological factors, which, by chance, would have acted synergistically to cause an increased risk of tick-borne diseases. Examples of national trends taken from a large collated, spatially explicit dataset [16, D. Sumilo 2006, *DPhil thesis*, Oxford University], to be published separately, substantiate this point (Fig. 8). While environmental awareness in Western Europe may have driven reduced industrial particulate pollution, possibly leading to the observed increased solar radiation (‘brightening’) and temperature increases [26], [27], in the post-communist Baltic States (and elsewhere) it was the collapse of industry that resulted in reduced pollution emissions. Indeed, a reversal from decreasing to increasing atmospheric transmission at the end of the 1980s has been recorded at Tartu, Estonia and at other sites around the world [27]. Alongside this, collective farming (both agricultural and livestock production) also collapsed at the beginning of the 1990s, leading to changes in land cover from agricultural and pasture to wooded land, with probable positive effects on the abundance of rodents (TBEv transmission hosts). At the same time, populations of wildlife hosts, particularly those such as deer, elk and wild boar that feed adult ticks, have fluctuated. The increase commonly recorded through the 1980s was abruptly, but temporarily, reversed in the early 1990s, possibly due to increased abundance of wolves as hunters turned their attention to more edible targets. A rare long-term record of a tick population in Rigas county, Latvia, indicates increasing abundance from the mid 1990s, but to levels no higher than those recorded in the 1970s, and rather too late to account fully for the c.3-fold greater incidence in TBE in Rigas county from 1993 (Fig. 2).

As the demand for labour in both agriculture and industry decreased in the Baltics, different approaches to managing this transition led to increased polarisation in economic conditions both between and within the countries during the 1990s. People in Estonia were more successful in keeping their jobs or moving into other sectors, especially in the growing service sector, than those in Latvia and Lithuania. In these latter two countries, more than half of those employed in agriculture in 1990 were not in employment by 2000, therefore relying on income from sources other than paid jobs (28).

A cross-sectional survey of over 1,000 people carried out in 2001 in Latvia showed that on average 69% of adults visited forests more or less regularly for work, food harvest or leisure purposes [29]. Nearly 80% of people in both the lowest and the highest income groups had visited forests over the previous 2 years, compared to 67% on average incomes, and were therefore more likely to be exposed to tick-infested habitats. Picking mushrooms and berries was stated as the principal reason for frequent visits to forests by female, older, poorer, less well educated and rural people, while younger people and the better paid and better educated city dwellers combined mushroom- and berry-picking with recreational walking on their less frequent visits [30]. Men were more likely to work in forests. Picking up a tick was not limited to, but was several times more likely amongst, forest visitors, and especially amongst people involved in food gathering or work. The role of human behaviour in determining risk of exposure to TBE virus is further emphasized by data on tick bites reported to Latvia's national Public Health Agency in Riga; irrespective of the local seasonal abundance of questing ticks, most tick bites occurred over the summer when rain-free weekends followed a week of heavy rainfall [30]. This pattern of exposure to ticks is consistent with people visiting forests to collect mushrooms immediately after good conditions for fungal growth.

Vaccination against TBE offers very effective protection, and people who had visited forests, no matter how infrequently, were more likely to have been vaccinated than those who had not visited a forest recently [29], [30]. Those on higher incomes, however, were up to five times more likely to be vaccinated than those on the lowest incomes. Therefore, even though both groups did indeed report higher than average contact with ticks, people on lower incomes would have had a higher probability of TBE infection if bitten by an infected tick.

Taking all factors into consideration, post-communist social transition would have created socio-economic conditions likely to increase human exposure to ticks to a greater or lesser extent. Variable contributions of each factor in different places at different times, due to normal regional variation in environmental and cultural conditions, alongside the more uniform changes in climate, would offer a powerful conceptual explanation for the spatio-temporal heterogeneity in upsurge in TBE across the Baltic States and also Central and Eastern Europe (see the EU-F6 EDEN project at http://www.eden-fp6project.net/), and also for the marked decreases in TBE incidence in many Baltic counties since 1999 [30]. Evidence for such variation exists and is highly suggestive (D. Sumilo 2006, *DPhil thesis*, Oxford University). The next goal is a spatially explicit quantification of this complex network of environmental and human factors, each changing independently yet indirectly linked, and apparently acting synergistically on the enzootic transmission cycles and/or degree of human exposure. This will allow predictions over large heterogeneous regions to test this hypothesis and warn of potential future changes if conditions continue to change in similar ways.

## Supporting Information

Table S1 Annual, monthly and dekadal (for April) mean daily maximum temperatures oC for 1989–2004 (columns A), and increases or decreases relative to 1970–88 (columns B). Statistically significant (p<0.05) changes are shown in bold.
(0.05 MB XLS)
